# Intimate Partner Violence: A New Cognitive, Interpersonal and Motivational Framework for the Rehabilitation of Perpetrators in Portugal

**DOI:** 10.1177/0306624X221148125

**Published:** 2023-01-12

**Authors:** Marta Capinha, Marco Pereira, Maria do Natal Sousa, Daniel Rijo

**Affiliations:** 1 University of Coimbra, Portugal; 2 General Directorate of Rehabilitation and Prison Services of the Portuguese Ministry of Justice, Portugal

**Keywords:** perpetrators program, court-ordered intervention, community-based intervention, early maladaptive schemas, motivational interview, schema-focused therapy, drop-out, recidivism, partner abuse, evidence-based recommendations

## Abstract

Intimate partner violence (IPV) has been targeted as a significant concern worldwide, but evidence for the efficacy of perpetrators’ interventions is not undisputable. This article briefly summarizes the evidence about perpetrators’ intervention efficacy, factors associated with recidivism, and evidence-based recommendations, before outlining the assumptions of a new community-delivered intervention aiming to rehabilitate IPV perpetrators: the CONTIGO Program. This program uses an innovative framework, focused on early maladaptive schemas, and combining cognitive, interpersonal, and motivational interview principles. The features of this intervention are discussed, and exploratory results regarding drop-out rates (8%) and recidivism (15.4%) in a sample of 162 court-mandated males are exposed. The detailed presentation of the CONTIGO Program and its intervention model represents a novel contribution that is sorely lacking in the IPV literature and could foster further research and debate about what can be done to effectively intervene with IPV perpetrators.

## Introduction

Intimate partner violence (IPV) has been targeted as a major concern across the world due to its high costs (European Institute for Gender Equality, 2014) and severe consequences to its victims (Hameed et al., 2020; Miller & McCaw, 2019; Stöckl et al., 2013). However, many questions remain regarding IPV interventions’ efficacy and the specific contents or strategies that may enhance their success.

Research focusing on the efficacy of IPV interventions has mainly assessed heterosexual couples in which women are identified as victims, probably because is the most prevalent form of IPV identified in legal systems (Hamel, 2007). Further violence toward intimate partners (whether based on partner or criminal reports) became the primary outcome when assessing IPV perpetrators’ intervention efficacy, as they aim for the reduction of recidivism rates (Lila et al., 2019). Recidivism rates for IPV are high, even when perpetrators are intervened, ranging between 15% and 60%, depending on how it is assessed and the follow-up period of the studies (Lila et al., 2019; López-Ossorio et al., 2017). Many studies ignored that a minimum of 1 year of follow-up period is recommended to prevent the overestimation of results (Dunford, 2000; Wilson et al., 2021), as the majority of IPV recidivism occurs during the first year after a formal IPV report (Lila et al., 2019).

Indeed, the efficacy of interventions for IPV perpetrators is yet to be established as mixed results have been systematically yielded in meta-analyses and literature reviews. For example, when examining criminal reports of new occurrences of IPV, meta-analytic findings (e.g., Babcock et al., 2004; Cheng et al., 2021; Davis & Taylor, 1999; Feder & Wilson, 2005) identified small positive effects of IPV perpetrators interventions. When using outcome data collected from victims’ reports in experimental studies, interventions seem to have very small, or slightly negative effects (Babcock et al., 2004; Cheng et al., 2021; Feder & Wilson, 2005). Differences in settings, length, treatment targets, and theoretical models, as well as some methodological limitations of the reviewed studies have contributed to the inconsistency of evidence about IPV interventions efficacy (Eckhardt et al., 2013; Henwood et al., 2015; Murphy & Ting, 2010). This remained unchanged, as the latest meta-analyses still struggle to find robust and coherent evidence of treatment efficacy (Karakurt et al., 2019; Nesset et al., 2019; Wilson et al., 2021), with positive medium effect sizes disappearing if couples’ reports are the source to assess recidivism (Arce et al., 2020), or losing statistical significance in follow-ups longer than 2 years (Travers et al., 2021). This grounded the call for exploring new interventions and new approaches to IPV interventions (Wilson et al., 2021).

Research on the efficacy of IPV interventions has also explored risk factors associated with recidivism itself. Variables such as being generally violent (not only within the family), the presence of family violence early in life, substance abuse problems, or trait anger, have been positively associated with recidivism (Lila et al., 2019; Shepard, 1992). Among these risk factors, drop-out from treatment has consistently emerged as a strong predictor, making its reduction one of the main challenges for IPV interventions (Lila et al., 2019; Olver et al., 2011). Drop-out rates have been found to range between 15%and 80% (Feder & Wilson, 2005; Lila et al., 2019; Olver et al., 2011; Shepard, 1992). These are concerning values, particularly if one considers that high retention rates (i.e., low drop-out) are a relevant indicator of intervention suitability (Babcock et al., 2004).

Proneness to recidivism may also be influenced by the existence of an active probation measure. Being under the supervision of the justice system would have a deterrent effect on criminal behavior and could influence outcomes regarding IPV interventions’ efficacy (Davis & Taylor, 1999; Maxwell et al., 2010; Murphy & Ting, 2010). For that reason, several authors recommended the description of court injunctions concomitant to treatment, and the duration of probation measures, when assessing IPV perpetrators’ interventions (Coker et al., 2002; Murphy & Ting, 2010). Finally, treatment dosage, and the elements of that treatment (e.g., addiction), may also impact recidivism. Hence, to allow for comparisons between the efficacy of different interventions they should be described in detail and include the identification of how treatment adherence was assessed (e.g., clearly defining what were the criteria for treatment completion) (Nesset et al., 2019).

### What do We Know About What Works?

So far, evidence has pointed to a few characteristics that seem to be associated with higher efficacy in interventions. A clear theoretical framework and well-defined structure with multimodal, skill-focused, and cognitive-behavioral therapy (CBT) community-based intervention have been found to be features contributing to effective offenders’ programs (McGuire, 2013). Also, the Risk-Need-Responsivity (RNR) model (Andrews & Bonta, 2017) has been found to contribute to intervention efficacy, including with IPV perpetrators (Stewart et al., 2014). This model highlights the importance of offering appropriate target interventions, aiming to change the offenders’ criminogenic needs, and tailoring interventions in face of individual differences and risk levels of offenders (Andrews & Bonta, 2017). RNR principles have been combined with other theoretical models when designing interventions, such as CBT, that have proven to be effective in aggressive behavior and offender rehabilitation (Lee & DiGiuseppe, 2018; McGuire, 2013). However, evidence for the efficacy of CBT interventions for IPV perpetrators is still inconclusive (Henwood et al., 2015; Nesset et al., 2019). It is important to notice, nonetheless, that what has been named CBT interventions for IPV, is not necessarily the same as CBT intervention in other areas of offenders’ treatment. Most addressed patriarchal beliefs and gender stereotypes to the point that became hardly distinguishable from the Duluth-inspired interventions (as these are their main targets), despite their conflicting assumptions (Babcock et al., 2004; Dutton & Corvo, 2007) and lack of empirical support for the efficacy of the latest (Arce et al., 2020; Babcock et al., 2007). Furthermore, patriarchal beliefs and gender stereotypes have been insufficient to explain IPV (Dutton & Corvo, 2007). Instead, empirical evidence has pointed to psychological variables (e.g., emotional dysregulation, early maladaptive schemas, attachment issues, personality traits) as more useful to understand and tackle it (Babcock et al., 2007; Corral & Calvete, 2014; Dutton & Corvo, 2007; Ehrensaft et al., 2004; Stuart, 2005). Despite this information, the need for promoting change on these variables, and going beyond automatic thoughts and/or cognitive distortions that are pro-violent (Bloomfield & Dixon, 2015; Hasisi et al., 2016), is usually overlooked.

Early maladaptive schemas (EMS), derived from Schema-Focused Therapy (SFT), have been pointed out as underlying IPV perpetrators’ aggressiveness (Pilkington et al., 2021; Rijo & Capinha, 2012). EMS arise from harsh life experiences that preclude children from having their emotional needs met (Rafaeli et al., 2011; Young et al., 2003). These experiences (e.g., physical, psychological, and sexual abuse), are known to be an important risk factor for IPV victimization (Coolidge & Anderson, 2002) and perpetration (Lila et al., 2019; Shepard, 1992). SFT (Rafaeli et al., 2011; Young et al., 2003) is an extension of CBT that integrates contributions from other models (e.g., attachment, developmental psychology). It has proven itself particularly helpful in treating severe interpersonal problems and personality disorders (Rafaeli et al., 2011), which have been identified in IPV perpetrators (Corral & Calvete, 2014; Fernández-Montalvo & Echeburúa, 2008). EMS guide the way people give meaning to relevant situations and organize how one copes with them, recreating the same self-defeating patterns throughout life. This is particularly striking in significant intimate relationships (Rafaeli et al., 2011; Young et al., 2003). Therefore, SFT may be an important contribution to unifying the existing literature on risk factors of IPV (Pilkington et al., 2021).

Because most interventions are delivered in forensic settings to court-ordered men, participants are not expected to be motivated to change or engaging intervention. This could help explain why motivational interview (MI) (Miller & Rollnick, 2002) has been another relevant feature consistently associated with better treatment outcomes in IPV interventions, including lower drop-out rates (Babcock et al., 2004; Musser & Murphy, 2009; Santirso et al., 2020). Randomized clinical trials have confirmed the positive effect of MI on IPV interventions (Alexander et al., 2010; Lila et al., 2018). The use of MI also seems relevant when substance abuse behaviors are present (Lila et al., 2020). Alcohol and drugs use have been identified as predictors of IPV and risk factors for recidivism due to their hypothesized disinhibitory effect on aggression (Caetano et al., 2005; Capaldi et al., 2012; López-Ossorio et al., 2017). Accordingly, addressing alcohol abuse problems in IPV interventions has also been recommended to enhance treatment efficacy (Caetano et al., 2005; Lila et al., 2020).

### The Present Work

Considering all the appeals and empirically driven recommendations discussed above, the main goal of the current paper is to present a new intervention for IPV perpetrators, combining cognitive, interpersonal, and motivational interview principles: the CONTIGO Program (Rijo et al., 2009). This is a governmental community-based response, implemented in the Azores Islands (Portugal) since 2009. It relies on an organized community network, actively participated by several stakeholders (police, prosecutors, the probation office, social welfare services, mental health services, victims support organizations, and other non-governmental agencies). The CONTIGO Program is strongly based on SFT (Rafaeli et al., 2011; Young et al., 2003) and includes the evidence-based recommendations reviewed above. It targets EMS and uses emotion-triggering activities in addiction to cognitive restructuring. The CONTIGO Program aims to overcome the limitations of “one-model-fits-all” interventions by adjusting itself to the participants’ identified needs (e.g., adding more sessions in the MI phase or referring the participant to specific intervention if alcohol abuse is present), as recommended by the RNR model. The detailed description of the program principles and intervention model stands as a relevant contribution to IPV literature, as programs tend to be vaguely presented, based on general and often misleading labels, as previously discussed. Although retrospectively, the current work also explores the CONTIGO Program participants’ recidivism and drop-out, as well as other proximal outcomes known to influence treatment response (namely, sociodemographic, probation-measure related, and dose-treatment related factors). Based on previous research, drop-out, younger age, presence of substance abuse/dependence, and lower treatment dosage were expected to be associated with recidivism. Nonetheless, the present study design does not allow for claims about the program effects, representing only an exploratory assessment to be followed by more robust investigation.

## Material and Methods

### Intervention Description

The underlying framework of the CONTIGO Program (Rijo et al., 2009) assumes that both victims and perpetrators have individual vulnerabilities—EMS/dysfunctional beliefs about the self—(individual variables) that interact with patriarchal beliefs and gender stereotypes (cultural variables), putting the basis for dysfunctional attitudes and behaviors to emerge within an intimate relational bond (relational variables). Differences in the attitudes and behaviors each person assumes in a violent intimate relationship would be influenced, not only by their EMS but also by the preferred coping mechanisms when a particular EMS is triggered and by cultural beliefs one may endorse. This theoretical approach states that while perpetrators tend to overcompensate for their EMS, victims tend to surrender to them. Overcompensation consists of doing the opposite of what one’s EMS makes us feel (e.g., attitudes of aggression, dominance, and manipulation are frequent) (Rafaeli et al., 2011; Young et al., 2003). It has been identified as a frequent coping style in general perpetrators (Brazão et al., 2017). In contrast, surrender means giving in to one’s EMS and accepting them throughout life. Dependence, passive behaviors, compliance, and dysfunctional ways of choosing a partner are some of the attitudes frequently identified as surrender coping styles (Rafaeli et al., 2011; Young et al., 2003). Hence, attitudes resulting from each type of coping style assumed to be typically used by victims and perpetrators are complementary, reinforcing the dysfunctional vision of the self, both in perpetrators and in victims. The relational bond between victim and perpetrator becomes the context that enables EMS to remain unchallenged and may even reinforce them. Finally, patriarchal beliefs and gender stereotypes, conceptualized as cognitive distortions, also contribute to the tendency to resort to these coping styles. They function as instrumental beliefs that allow the aggressive behavior to be exhibited and justify it, reducing perpetrators’ shame and/or guilt before and after aggression, or supporting victims’ difficulties to leave dysfunctional and harmful relationships. This theoretical framework aims at a comprehensive view of the dynamics of IPV, recognizing its relational context. Nevertheless, the intervention that derives from it does not diminish the perpetrators’ responsibility for their behavior.

Indeed, the main goal of IPV interventions is to prevent recidivism and contribute to the victim’s security. To accomplish that, the CONTIGO Program aims to reduce the interference of EMS in social information processing, which may enhance aggressive behaviors in an intimate relationship. At the same time, it aims to overcome the resort to patriarchal beliefs and gender stereotypes to legitimate that behavior and its recurrence.

The CONTIGO Program includes different phases and modalities: (1) an initial individual MI intervention; (2) a CBT structured group program (SFT-inspired); and (3) a final individual MI intervention. It also allows an optional intervention phase for couples and the referral for complementary interventions according to participants’ needs. The initial MI intervention consists of individual sessions aiming to decrease the resistance to change and increase treatment adherence. The number of sessions depends on the participant’s needs and can co-occur during the structured group sessions, usually with lower frequency. If there is any evidence of drug or alcohol abuse or mental health issues, participants are sent to a specialized intervention until any addictive behavior or mental health symptoms are considered stable enough to start the next phase.

The next phase is the CBT structured group program. It is an open-ended program made of 18 sessions each lasting about 90 minutes, which runs weekly. Sessions must be carried out by two facilitators, and at least one is a psychologist skilled in CBT, SFT, and MI. Each session is devoted to a theme: (1) gender stereotypes; (2) gender stereotypes and interpersonal relationships; (3) sadness; (4) fear; (5) guilt and apology; (6) shame; (7) intimacy; (8) anger and aggression; (9) coercion; (10) self-concept; (11) inferiority and failure; (12) emotional deprivation and dependence; (13) entitlement; (14) insecurity, jealousy, and control; (15) my partner qualities; (16) destructive criticism and humiliation; (17) compliments and appreciation for others, and (18) communication/negotiation. Sessions 1 and 2 are targeted at cultural beliefs. Their goals are to understand and recognize gender stereotypes and their influence on the behavior toward others and the self, to acknowledge that stereotypes may be harmful to both women and men, and to challenge them. Individual variables are the target of sessions that address emotions and EMS. Sessions focused on emotions are important because emotions associated with EMS tend to be overwhelming and misguided (Rafaeli et al., 2011). These sessions aim to identify the body sensations, thoughts, and behaviors usually associated with the emotion, to understand the emotions’ evolutionary roles, to recognize when each emotion may become dangerous or damaging for the self and others, and to practice their proper expression. Sessions 10 to 14 focused directly on EMS. These are designed to make the EMS less prominent, trying to diminish the number of situations (and intensity) in which they are triggered. Sessions aim to understand the concept of EMS, to recognize their influence on one’s emotions and behavior, and to practice alternative ways of thinking/experiencing situations to counterattack these EMS. Finally, relational variables are worked on in the remaining sessions, aiming to identify potentially dysfunctional attitudes and behaviors in the intimate relationship, understand their advantages and disadvantages to the self, and develop alternative attitudes and behaviors that better serve the purpose of a healthy intimate relationship.

All sessions have a similar structure, starting with an overview of the previous session and sharing insights or related events that occurred during the week. The theme of the session is then presented throughout a triggering exercise, followed by a guided debate, where participants’ beliefs and attitudes are challenged. MI principles are always observed. The debate ends with a role-play activity in which participants are prompted to act or express themselves in a more adjusted way. In the end, a synthesis of the session is made, and a “Trump Card” is given to each participant; this card displays a key phrase that summarizes the session and serves as a reminder of the “homework”: to practice the contents of the session in real-life situations.

After the structured group program, the individual MI intervention is reinforced again, with a focus on relapse prevention and risk management. In this stage, a completely volunteered phase of couples’ therapy is available for those couples who remain together or for participants engaged in new intimate relationships. The goals are defined with each couple and could vary from improving the couple’s communication to intimacy issues.

### Participants

Between January 2009 and July 2016, 225 perpetrators were identified as being court-ordered to an IPV intervention in the Azores Islands where the CONTIGO Program was delivered. Participants must have completed probation measures at least 2 years ago to be eligible for this study. This limited the possible influence of the justice system supervision over recidivism and surpassed the recommendation for observing the first year after the first offense (Wilson et al., 2021). From this initial sample, five registries were found to be duplicated, one subject died before any intervention, seven refused to adhere to the probation-measure supervision, one was a female, and 39 participants did not fulfill the CONTIGO Program eligibility criteria and were excluded from the analyses. Reasons for exclusion were: 13 subjects had cognitive impairment (the program is not suitable for those cognitively impaired); nine had severe psychiatric disorders (the experiential exercises used in the program are not suitable for patients suffering from psychotic-spectrum disorders); 11 showed severe or multiple personality disorders (a population more resistant to intervention, with specific intervention needs) and six exhibited severe substance abuse. Ten subjects from this initial sample (*N* = 225) were also excluded from the analyses because they were drop-outs from the probation measures (meaning that they fulfilled the eligibility criteria to initiate intervention but were suspended by court order due to probation violation). Of these 10 participants, four were suspended due to recidivism. They were not included in the recidivism rates after intervention once it was not possible to determine the time of the recidivism (i.e., the re-offense may have happened even before the application of the probation measure while awaiting trial for the first offense). Furthermore, as some authors stressed (e.g., Babcock et al., 2004), if the outcome data were based on “intention to treat” it could be biased as participants are being assessed without having received an adequate dose of treatment.

The final sample consisted of 162 IPV perpetrators, with a mean age of 41.25 years (*SD* = 9.78: range: 21–71 years), and a mean of 5.56 school years (*SD* = 2.22) (see Table 1). For those who were in an intimate relationship with the victim at the beginning of the intervention (75%), the average relationship length was 15.14 years (*SD* = 9.83). Probation measures were applied according to Portuguese law, with an average of 18.01 months of duration (*SD* = 8.94).

**Table 1.** Sociodemographic Characteristics of the Sample (*N* = 162).

	*N*	%
Socioeconomic status		
Low	126	77.8
Medium	36	22.2
Marital status		
Single	2	1.2
Married	55	34.0
Cohabiting as husband and wife	20	12.3
Divorced	33	20.4
Separated	41	25.3
New relationship	11	6.8
Maintain an intimate relationship with the victim after the intervention		
Yes	65	40.1
No	96	59.3
No information	1	0.6
Probation measure		
Applied before trial	112	69.1
Applied after conviction	50	30.9
Structured intervention setting		
Group	135	83.3
Individual	27	16.7

*Note*. The CBT structured group program has its sessions adapted to be conducted in an individual format, due to the reduced number of participants in smaller islands, which does not allow a group to be formed.

### Measures

The primary outcome of this study is the recidivism rate of IPV 2 years after the end of the probation measure. It was calculated based on any new charges of an IPV-related crime presented in police departments, against the same or a new partner. This included actual, attempted, or threatened violence (physical, sexual, psychological, or other). Recidivism was considered, regardless of whether there was an arrest or a conviction. Drop-out rates and treatment doses were also analyzed as secondary outcomes, and other injunctions concomitant to treatment were described. Participants were considered drop-out from intervention if they miss four sessions. If a participant misses up to three sessions, he will be allowed to do those sessions at another moment. Completers must have attended 100% of the program sessions.

### Procedures

This is a retrospective study with male IPV perpetrators who completed the CONTIGO program between 2009 and 2016. Data were collected from police and probation office criminal records. It was possible to ensure that no data of the identified participants were lost due to obstacles in data tracking on police records. All procedures were per the ethical standards of the Ethics Committee of the university, police, and probation office.

### Data Analysis

Data analyses were conducted using the IBM SPSS 20 software. Participants were coded for the existence or not of IPV recidivism. A multiple logistic regression analysis was performed to explore the factors (sociodemographic, probation-measure related, and dose-treatment related) associated with recidivism status (re-offender or not re-offender). Variables with a *p*-value < .25 in the univariate analysis were included in the multivariate analysis with backward selection and displayed as odds ratios (OR) and confidence intervals (CI). The model’s goodness of fit was measured with the Hosmer-Lemeshow test.

## Results

### Treatment Dose and Court Injunctions or Rules of Conduct

In addition to the CBT structured group program, 63.6% (*n* = 103) of the participants were sent to specialized intervention due to addiction behavior, and 17.3% (*n* = 28) due to mental health symptoms. Couples therapy was completed by three participants (1.9%). Participants completed an average of 14.41 sessions of MI (*SD* = 11.35; missing responses = 3) during the probation measure, and most of the participants (62.8%, *n* *=* 100; missing responses = 3) made between 6 and 15 sessions. Among the records available, it was not possible to discriminate the number of sessions from the first and the last phase of the MI, nor the number of sessions of the other complementary interventions. Participants were also ordered to observe other court injunctions/rules of conduct: 4.9% had regular police supervision, 9.2% were forbidden to have any contact with or to get near the victim, and 1.2% were forbidden to use or possess any firearms. Other injunctions were applied (e.g., payment of compensation to the victim) in 8.6% of the cases.

### Drop-out From Intervention

A rate of 8% was found regarding drop-out of the structured intervention phase after three participants (1.9%) previously excluded by missing sessions re-enroll the intervention by court order and completed it. Drop-outs from the intervention were mainly due to missed CBT structured group program sessions (*n* = 12, 7.4%), but 2.5% (*n* = 4) were drop-outs for other reasons (one immigration with court authorization; two expulsions for disciplinary reasons; one conclusion of the probation measure during intervention).

### Recidivism

The study shows that the IPV recidivism rate during the 24 months of follow-up was 15.4%, divided by 12 participants (8.0%) that had reports only for IPV recidivism, and 11 (7.4%) that had reports for IPV recidivism concurrently to reports for other crimes. Most participants (*n* = 111; 74.5%) do not have any report (for IPV or other crimes) and 15 (10.1%) had reports for other crimes, but not IPV. Univariate analyses did not identify any significant variable associated with IPV recidivism. In the multivariate model, none of the assessed variables were found to be significantly associated with recidivism (Table 2). The final logistic regression model was not significant, *χ*^2^ (4) = 7.98, *p* = .092.

**Table 2.** Univariate and Multivariate Logistic Regression Analysis of the Association Between Sociodemographic, Probation-Measure Related and Dose-Treatment Related Variables and Recidivism Status.

Variables	Univariate analyses		Multivariate analyses	
	OR [95% CI]	*p*	OR [95% CI]	*p*
Age	1.00 [0.96–1.05]	.974		
Education years	1.05 [0.86–1.27]	.636		
Relationship status	1.98 [0.80–4.91]	.141	2.07 [0.79–5.40]	.137
Relationship duration	1.00 [0.99–1.00]	.946		
Probation measure type	0.99 [0.38–2.59]	.979		
Number of MI sessions	1.03 [0.99–1.06]	.113	1.02 [0.99–1.06]	.194
Structured program setting	0.41 [0.09–1.85]	.243	0.46 [0.10–2.22]	.338
Addiction behavior treatment	1.31 [0.50–3.43]	.577		
Psychiatric/psychological intervention	2.19 [0.80–5.97]	.127	2.58 [0.90–7.37]	.077

*Note.* Only covariates associated with recidivism (two-sided *p*-value < .25) are shown and included in the multivariate logistic regression model. OR *=* odds ratio; 95*%* CI = 95% confidence interval. Dependent variable: 0 = no recidivism, 1 = recidivism; Covariates: relationship status = relationship with the victim at the beginning of the probation measure (0 = not in relation; 1 = in relation), probation measure type (0 = before trial, 1 = after trial), structured program setting (0 = group vs. 1 = individual), addiction behavior treatment (0 = no, 1 = yes), psychiatric/psychological intervention (0 = no, 1 = yes).

## Discussion

There is a growing number of interventions for IPV perpetrators available across the world. The current work provides a brief and up-to-date summary of evidence about perpetrators’ intervention efficacy, factors associated with recidivism, and evidence-based recommendations derived from these findings. Despite considerable research being made, questions about IPV intervention efficacy remain without a definitive answer. Further research in the field is being encouraged (Cheng et al., 2021), including new approaches to intervention (Wilson et al., 2021). Therefore, the main goal of the present work is to provide a detailed description of the intervention delivered by the CONTIGO Program, and the characteristics potentially associated with treatment failure. This is, to the best of our knowledge, the first paper to identify participants’ recidivism after a community-based intervention for IPV perpetrators in Portugal.

The CONTIGO Program is based on a conceptual model that includes individual, relational, and cultural variables, deemed as necessary to overcome the limitations pointed to most interventions (e.g., Babcock et al., 2007; Bowen, 2011; Dutton & Corvo, 2007). The CONTIGO Program multimodal approach allows targeting specific criminogenic needs identified for each IPV perpetrator, recognizing that “one-model-does-not-fit-all” (Bowen, 2011). The structured intervention follows the guidelines for effective interventions with offenders (Andrews & Bonta, 2017; McGuire, 2013). The fact that this is a community-based program can be regarded as an advantage, considering it is cost-efficient and less detrimental to participants’ social and professional relationships, not adding additional risk factors to their pathways. Following the recommendations of several authors (e.g., Cheng et al., 2021; Nesset et al., 2019), this work described all treatment components of the CONTIGO Program and all the concomitant injunctions/rules of conduct that participants were obliged by the court. This aims to facilitate future comparisons with other interventions.

The recidivism rate found for CONTIGO Program completers was 15.4%. The observed recidivism is lower than the average recidivism rates found in efficacy studies with a similar design (26%) (Davis & Taylor, 1999), and other CBT interventions with 2 years follow-up (ranging from 25.5% to 42.5%) (Bloomfield & Dixon, 2015; Scott et al., 2015). Nonetheless, it is higher than official recidivism found in more recent studies, ranging from 6.25% to 8.75% (at 6 months follow-up; (Lila et al., 2018), or 7.38% (1 year follow-up) (Lila et al., 2019). Unfortunately, the descriptive nature of the current study does not allow for conclusions to be drawn from these comparisons. Nonetheless, it provides an important reference point that may be useful for other researchers wanting to assess IPV recidivism in Portugal.

Regarding secondary outcomes, the drop-out rate of 8% is one of the lowest found in the literature (Feder & Wilson, 2005; Olver et al., 2011), including when compared to other interventions that use also MI and present lower recidivism rates (after 6 months of follow-up) (Lila et al., 2018). This finding is encouraging, particularly because the definition of treatment completion implies that participants should attend all the structured group program. Therefore, 92% of the participants completed 100% of the structured program sessions, suggesting that the CONTIGO Program is appropriate for the targeted population (Babcock et al., 2004). It could also indicate that the use of MI is useful at building and sustaining motivation, ultimately contributing to prevent drop-out. To be noticed that being a community-based intervention, the CONTIGO Program excludes the perpetrators with the highest risk and with more antisocial characteristics, since these are supposed to be incarcerated by Portuguese law. Future research should include a formal assessment of perpetrators’ risk and characteristics once they could imply different probabilities of recidivism and drop-out (Lila et al., 2018).

Concerning the treatment dose, it is to be noticed that most participants needed substance abuse/dependence treatment. This is in accordance with the literature about risk factors for IPV, which indicates that substance abuse/dependence (mainly alcohol) is frequently found in IPV perpetrators (López-Ossorio et al., 2017). Substance abuse is also associated with IPV recidivism, as well as the presence of mental health symptoms (especially depressive symptoms), age (the oldest the perpetrator, the lower the risk), among other factors (Capaldi et al., 2012; Lila et al., 2019). However, none of the analyzed variables revealed itself as a significant predictor of recidivism. Future studies should continue to explore these associations in larger samples, as well as other recidivism predictors already found in the literature but not considered in this work (e.g., Lila et al., 2019).

Taking into consideration that these are only preliminary findings, several limitations should be acknowledged, most importantly the lack of a randomized controlled design with a control or comparison group. This non-experimental design strongly limits the conclusions that can be drawn about the CONTIGO Program efficacy and prevent treatment effect size calculation. Future research including a randomized controlled design is required before establishing the CONTIGO Program’s efficacy. Sources other than police records (e.g., victims’ reports) should also be included in future research, once effect sizes of IPV interventions tend to disappear if victims’ reports are considered (Cheng et al., 2021). Future studies should also focus on relevant variables related to the CONTIGO Program theoretical assumptions (e.g., EMS). Investigating the potential program effects on those variables would be crucial to understand the mechanisms behind an eventual cognitive, emotional, and behavioral change in participants. A more systematic evaluation of the processes and variables involved in change observed in participants could further clarify what processes lead to higher treatment adherence, and more effective and durable outcomes. Future research could also include feminine perpetrators.

Research regarding the CONTIGO Program efficacy is still an ongoing process, but the present findings offer preliminary support for the CONTIGO Program as its retention ratio is higher than expected, given previous findings. For the same reason, they also encourage the implementation of MI to deal with treatment engagement and retention issues within IPV perpetrators.
